# Time Perspective and Age: A Review of Age Associated Differences

**DOI:** 10.3389/fpsyg.2017.00101

**Published:** 2017-02-17

**Authors:** Daniella Laureiro-Martinez, Carlos A. Trujillo, Juliana Unda

**Affiliations:** ^1^Department of Management, Technology and Economics, ETH ZurichZurich, Switzerland; ^2^School of Management, Universidad de los AndesBogota, Colombia

**Keywords:** time perspective, age, meta analysis, goal setting, socioemotional selectivity theory

## Abstract

We investigate the relationship between age and the five dimensions of time perspective measured by the Zimbardo Time Perspective Inventory (ZTPI) (past negative, past positive, present hedonistic, present fatalistic, and future). Time perspective is related to well-being, decision-making, level of development, and many other psychological issues. Hence, the existence of a systematic relationship between time perspective and age should be considered in all studies for which time is a relevant variable. However, no specific research about this has been conducted. We collected 407 papers that referenced the ZTPI between 2001 and 2015. From those, 72 studies met our inclusion criteria. They included 29,815 participants from 19 countries whose age spans most phases of adulthood (from 13.5 to 75.5 years, mean 28.7). We analyzed these studies adapting meta-analytical techniques. We found that present hedonistic and past negative dimensions are negatively related to aging with partial eta squared effect sizes of roughly 0.15. Our results have implications for the design of studies related to time as our findings highlight the importance of taking into account the differences associated with age.

## Introduction

People’s views of themselves, their world, their goals, and their relationships are filtered through temporally based cognitive processes (Keough et al., 1999). The temporal categories under which individuals classify their experiences play a fundamental role in the selection and pursuit of social goals, with important implications for emotional, cognitive, motivational, and social processes (Zimbardo and Boyd, 1999). Time perspective is the outcome of a “process whereby the continual flows of personal and social experiences are decomposed or allocated into temporal categories” (Zimbardo and Boyd, 1999, p. 1271). Time perspective is a pervasive construct that is present in our way of conceiving the world and affects a myriad of behavioral outcomes. The Zimbardo Time Perspective Inventory (ZTPI), created in 1999, is the most used measure of time perspective. A very extensive body of empirical data based on the ZPTI has shown that time perspective is used in encoding, storing, and recalling experienced events during life, as well as in forming short and long term goals. For instance, well-being is more influenced by time perspective than by the Big Five personality factors (Zhang and Howell, 2011). Time perspective also predicts multiple fundamental life factors such as health, happiness, and financial and environmental behaviors (Cunningham et al., 2015; Hall et al., 2015; Klicperová-Baker et al., 2015; Milfont and Christophe, 2015).

Time perspective has been characterized as a relatively stable individual view that likely evolves with age (Zimbardo and Boyd, 1999). However, little is known about how and which perspectives change through life-span stages. Understanding how time perspective changes through life is important, as different realms of human psychology (e.g., development, decision making, goal setting, and personality) are potentially influenced by both temporal categorization and age. The existence of a systematic relationship between the two variables would engender moderating effects that would go unnoticed by studies that use samples with limited age ranges. Therefore, such studies may fail to identify age specific results. Not surprisingly, several authors have made calls to better understand if and how time perspective is different at different ages (Nurmi, 2005; Mello and Worrell, 2006; Matthews and Stolarski, 2015). This paper contributes to answering those calls.

Although studying age and the five dimensions of time perspective has not been the specific focus of past studies, some have reported the effect of age as a covariate of some of the dimensions. Findings are, however, inconclusive. There are various reasons for such ambiguity: specificity of the samples’ population, variability in the age ranges, different gender compositions, different research designs, and limited sample sizes. A summary of different studies that further exemplifies the lack of clarity is presented on pages 521–538 of Stolarski et al. (2015a).

Why should time perspective be different at different life stages? According to socioemotional selectivity theory^1^ (SST), aging can be understood as a process of learning and adaptation (Carstensen et al., 2011). SST findings propose that the perception of time plays a fundamental role in the selection and pursuit of social goals, with important implications for emotion, cognition, and motivation. The Strengths and Vulnerability Integration model (SAVI) proposes two mechanisms that could explain so (Charles, 2010). One, the perception of the amount of time left in life increases the prioritization of emotion-related goals. As individuals age, they pay more attention to emotional goals which take precedence over the knowledge ones (Carstensen et al., 1999; Carstensen, 2006). Two, the time lived allows for the necessary experience to better navigate daily life. As individuals age, they learn and emotionally regulate the responses to the environment (Lee et al., 2016)

We rely on these theories and their findings to develop expectations on the relationship between age and time perspective. We expect to observe a pattern of adaptation to life such that older people focus more on emotional well-being. Given that the five time perspectives are independent, meaning that people can shift their attention among different time periods and that focusing on one perspective does not necessarily prevent thinking about the other perspectives, we create independent hypotheses for the past, the present and the future without erroneously assuming that what affects one of the times would necessary affect the others.

First, the past negative perspective refers to the tendency to look back on life with regret, ruminating on the bad things that have happened in the past, while the past positive perspective refers to looking fondly, almost with ecstasy, on the past events. There is mounting evidence to support the idea that older people tend to remember the past more positively than younger people (Levine and Bluck, 1997; Carstensen and Löckenhoff, 2003; Charles et al., 2003; Lockenhoff and Carstensen, 2003; Mather and Carstensen, 2005; Carstensen et al., 2006; Isaacowitz et al., 2006; Kim et al., 2008). As individuals grow older, their past memories become tinted with more positive and less negative tones. This could be translated into both a positive relation between age and past positive, and a negative relation between age and past negative.

Second, the present hedonistic perspective is associated with a self-indulgent view of current events, while the present fatalistic is associated with a passive and stoic interpretation of them. Various prominent models of development have proposed the idea that as people get older they draw on their accumulated expertise and become more selective in their goals and social relations, prioritizing their emotional goals (e.g., Baltes and Baltes, 1990; Carstensen et al., 1999; Baltes et al., 2006). As individuals grow older, they will, on average, lower their resignation attitudes and gain more control of their lives, diminishing the belief that they are at the “whimsical mercy of ‘fate”’ (Zimbardo and Boyd, 1999 in 2015 p. 34). In addition, as individuals grow older they can draw on their past experiences’ outcomes to gain a more internal locus of control (Peterson and Stunkard, 1992), diminishing their sensation and risk-seeking propensities, and reducing what Zimbardo and Boyd called the “the devil may care” attitude^2^. One could then expect a negative relation between age and present fatalistic and present hedonistic.

Finally, the future perspective refers to the depth of attention and consideration given to future events. During youth, time is perceived as expansive, and long-term goals are chosen over others because they optimize future possibilities – for example, children imagine the profession they will follow. However, as we age, time is perceived as limited, and short-term goals, such as social connectedness, social support, and emotional regulation assume highest priority (Carstensen et al., 1999). During childhood, the cognitive abilities involved in planning for the future are not yet fully developed; in adolescence and early adulthood such abilities are in place. Strough et al. (2016) found that people focused more on future opportunities than on the fact that time is limited through middle age, and then shifted to focusing less on future opportunities and more on time limitations at around 60 years of age. However, it seems that what varies is the content, that is the patterns of goals and expectations that different age groups tend to focus on (Seginer, 2009). Since what changes is the set of age-specific goals people attend to, we do not expect age to change future time perspective.

## Method

To explore the relationship between age and time perspective we used meta-analytic techniques and looked at studies that used the ZTPI and reported the age of their participants. We searched for published and unpublished studies that, independently of their main topic, reported results about age and time perspective from the ZTPI (Zimbardo and Boyd, 1999).

### Selection Criteria

We selected studies that contained age information and details about the sample population. We only included studies that used professional translations of the inventory. We excluded studies that used previous, abridged or incomplete versions (For example: Mckay et al., 2015). Since multiple authors have identified time perspective as a stable individual trait not affected by the experimental setting (Zimbardo and Boyd, 1999), we did not filter studies based on the contextual manipulations they used but we control for those as for the language of the instrument. Since the first study that meets our criteria was published in 2001, we limited our search to studies published from 2001 to 2015.

### Search Strategy

Published studies were identified by two research assistants on Scopus and PsycINFO (three waves: October 2013, July 2014 and April 2015). Search terms were “Zimbardo Time Perspective Inventory,” “ZTPI,” “Time Perspective,” and “Zimbardo.”

Unpublished studies were identified by calls on the major listservs in social psychology, on the Time Perspective Network listserv, social media accounts and by direct requests to 91 authors who have done research using the ZTPI and to eight authors who have developed tests that measure individual time propensities (wave December 2016). In addition we also searched for unpublished master and doctoral theses.

An overview of the search strategy and the results at each stage is depicted in **Supplementary Figure S1**. From the published data sources, we identified 400 published papers that potentially met the selection criteria. We excluded 294 papers because they used the ZTPI only to theorize. From the calls for unpublished data, we obtained seven studies. After an in-depth review, we further excluded 37 published and four unpublished papers that did not report information about the sample or the means of the different time perspectives.

The final database included 72 studies included in 52 papers spanning 19 countries (See **Supplementary Table S1**). From these, 57 published and three unpublished studies report all five ZTPI perspectives. Twelve published studies report one or two of the ZTPI perspectives. The total number of participants included in these studies was 29,815. The mean age was 28.7 and ranged from 13.5 to 75.5 years, which, importantly, spans most phases of adulthood. There was, however, more representation of adolescents and young adults in the sample than middle-aged and older adults: 33% of the sample studies had a mean age below 21 years, 42% between 21 and 35, 19% between 35 and 50 and 6% over 50. Nonetheless, this allows us enough range of years to observe changes in time perspective attributable to different life stages. According to our theorizing, changes in time perspectives occur in the long term and are associated with life stages, hence studies conducted with participants with a narrow age range (i.e., people in the same life stage) should not report a relationship.

## Analyses

As expected, we found that single studies tend to target populations with narrow age ranges which render any reported relationship incomparable across studies. **Supplementary Figure S2** contains the mean age vs. the age standard deviation for all selected studies, showing the narrow age range of individual studies. There is a positive relationship between mean age and standard deviation (*r* = 0.63; *p* < 0.00).

We looked for the relationship between age and time perspective, within the selected studies, to be explicitly reported so effect sizes can be obtained. However, we found within the selected sample of studies that this information is absent. In spite of age being frequently included as a covariate in regression analyses its relationship (i.e., a regression coefficient or estimated effect) with time perspective is rarely reported. Regarding the possibility of publication bias in the relationship between age and time perspective, our search for published and unpublished works resulted in a high number of published studies that do not address this question directly. They only contain data on time perspective and age while studying other issues. This is consistent with the absence of effects being reported and the very low number of unpublished works that we obtained through the usual channels. Therefore, there are reasons to suspect that publication bias is not probable for our review and we could not obtain enough unpublished data to assess it empirically.

To explore the relationship between age and the different time perspectives, we estimated a random effects meta-regression (Borenstein et al., 2009) for each of the five time perspectives scores using mean time perspective scores instead of effect sizes and time perspective standard deviations instead of the standard errors of the dependent variable. This is an adaptation of regular meta-regression analysis that allows us to test the relationship of interest. It includes a test for heterogeneity (*tau^2^*) among studies regarding the estimated time perspective means while still taking into account that means are estimates whose dispersion is captured by the standard deviation. Effect sizes are also estimates whose dispersion is captured by the standard error. The main independent variable is mean age. This adaptation changes the interpretation of the meta regression results such that the estimated marginal effects are a direct measure of the effect size. In standard practice, the dependent variable is composed by effect sizes, hence the estimated effects of the independent variables are average changes in the effect sizes. This adaptation may constitute a methodological contribution that extends the standard use of meta-regression. To complement the analysis and improve clarity, we also ran standard linear regressions in order to have a robustness check and to obtain meaningful measures of effect sizes such as partial eta squared. In all analyses we included relevant covariates: year of publication, whether the study was conducted in a neutral or a special context (dummy coded), whether there was a prior manipulation (dummy coded) and the percentage of women in the study.

## Results

**Table 1** presents the meta-regression results^3^ and **Table 2** presents the linear regression results. Bold values highlight significant coefficients. We found a negative relationship between age and time perspective for both the Past Negative and the Present Hedonistic time perspectives that is consistent across regression methods: for both time perspectives the average scores decrease as the samples are older, indicating that older people are less negative about the past and less hedonistic. The meta regression suggests that this relationship is slightly stronger for Present Hedonistic but partial eta squared effect sizes calculated from the standard linear regressions reveal the effect sizes to be similarly large (around 15% of variance). Chi-squared tests also show that the marginal effects of present hedonistic and past negative are not significantly different from each other using estimations from meta regressions (χ*^2^* = 0.14; *p* = 0.7) and linear regressions (χ*^2^* = 0.05; *p* = 0.8) Tau^2^ is close to 0 for all models, meaning that there is very low between-studies variance. Including or excluding the three unpublished studies in our data base has no effect on the results.

**Table 1:** Meta-regressions of mean study age on mean time perspective scores.

	Past negative			Past positive			Present fatalistic			Present hedonistic			Future		
					
	Coeff	(*SE*)	*p*(z)	Coeff	(*SE*)	*p*(z)	Coeff	(*SE*)	*p*(z)	Coeff	(*SE*)	*p*(z)	Coeff	(*SE*)	*p*(z)
Sample mean age	**-0.01**	**(0.00)**	**<0.1**	-0.00	(0.00)	ns	0.00	(0.00)	ns	**-0.02**	**(0.00)**	**<0.01**	0.00	(0.00)	ns
Year of publication	**0.05**	**(0.02)**	**<1**	**0.10**	**(0.02)**	**<0.01**	**0.07**	**(0.02)**	**<0.01**	**0.11**	**(0.02)**	**<0.01**	**0.10**	**(0.02)**	**<0.01**
Percentage of females	-0.85	(0.55)	ns	0.03	(0.48)	ns	-0.47	(0.47)	ns	-0.34	(0.40)	ns	0.09	(0.51)	ns
Context of application	0.20	(0.28)	ns	-0.02	(0.24)	ns	0.07	(0.23)	ns	0.07	(0.41)	ns	0.05	(0.24)	ns
After other questionnaires	-0.04	(0.19)	ns	-0.18	(0.16)	ns	-0.21	(0.15)	ns	-0.19	(0.13)	ns	-0.16	(0.16)	ns
Questionnaire modification	-0.15	(0.44)	ns	-0.18	(0.38)	ns	0.10	(0.20)	ns	0.12	(0.17)	ns	0.06	(0.21)	ns
Constant	**-94.6**	**(52.6)**	**<0.01**	**-217**	**(0.48)**	**<0.01**	**-139**	**(44.3)**	**<0.01**	**215**	**(36.5)**	**<0.01**	**-203**	**(48.1)**	**<0.01**
Tau2	0			0			0			0			0		
Number of studies^a^	56			56			66			66			67	


*^a^N’s change because of missing data.*

**Table 2:** Linear regressions and effect sizes of mean study age on mean time perspective scores.

	Past negative				Past positive				Present fatalistic				Present hedonistic				Future			
					
	Coeff	(*SE*)	*p*(t)	P. Eta2	Coeff	(*SE*)	*p*(t)	P. Eta2	Coeff	(*SE*)	*p*(t)	P. Eta2	Coeff	(*SE*)	*p*(t)	P. Eta2	Coeff	(*SE*)	*p*(t)	P. Eta2
Sample mean age	**-0.01**	**(0.00)**	**<0.01**	**0.15**	-0.00	(0.00)	0.266	0.02	-0.00	(0.00)	0.497	0.00	**-0.01**	**(0.00)**	**<0.01**	**0.16**	-0.00	(0.00)	0.755	0.00
Year of publication	**0.03**	**(0.01)**	**0.027**	**0.09**	**0.09**	**(0.02)**	**<0.01**	**0.27**	**0.03**	**(0.01)**	**0.018**	**0.09**	**0.06**	**(0.01)**	**<0.01**	**0.28**	**0.07**	**(0.02)**	**<0.01**	**0.11**
Percentage of females	**-0.66**	**(0.27)**	**0.019**	**0.10**	0.17	(0.37)	0.659	0.00	-0.32	(0.29)	0.275	0.02	-0.13	(0.26)	0.626	0.00	0.20	(0.49)	0.684	0.00
Context of application	0.17	(0.13)	0.203	0.03	-0.00	(0.19)	0.963	0.00	0.03	(0.14)	0.813	0.00	-0.00	(0.13)	0.951	0.00	-0.15	(0.24)	0.527	0.00
After other questionnaires	-0.04	(0.09)	0.644	0.00	-0.11	(0.14)	0.422	0.01	**-0.20**	**(0.09)**	**0.046**	0.06	-0.15	(0.08)	0.095	0.04	-0.04	(0.16)	0.784	0.00
Questionnaire modification	-0.13	(0.21)	0.536	0.00	0.08	(0.30)	0.792	0.00	-0.00	(0.13)	0.950	0.00	0.07	(0.12)	0.540	0.00	0.09	(0.21)	0.664	0.00
Constant	**-63.7**	**(29.6)**	**0.036**		**-179.1**	**(42.1)**	**<0.01**		**-73.3**	**(0.31)**	**0.023**		**-132.3**	**(28.4)**	**<0.01**		**-142.9**	**(52)**	**<0.01**

*R*^2^	0.28			*p*(F)	0.36			*p*(F)	0.18			*p*(F)	0.46			*p*(F)	0.14			*p*(F)
				0.010				<0.01				0.023				<0.01				0.141

Number of studies^a^	56				56				66				66				67


*^a^N’s change because of missing data.*

Regarding covariates only, the year of the study’s publication has a significant and positive effect on all time perspective dimensions, meaning that recent studies tend to report higher values on all the time perspective dimensions. This effect may be attributable to event-related cohort effects that may have affected the whole sample. However, our sample included one paper with three studies that reported unusually low time perspective scores and was published before 2002. After removing these three studies the effect of year disappears, while the effects of age remained significant and effect sizes are similarly large (see **Supplementary Figures S3**–**S6** for the evidence on this issue). The other covariates display no relationship with estimated time perspective scores.

## Discussion

Our cross-sectional age comparisons point to the possibility that there is a marked improvement in emotional experience from early adulthood into old age, and reaffirm and complement the ideas proposed by the SST and SAVI. In short, our main finding is that older individuals appear to focus less on the negative events of the past, and approach the present in a less hedonistic manner.

Our evidence is consistent with the notion that as individuals grow older, their past memories become tinted with less negative tones (Mather and Carstensen, 2005; Carstensen et al., 2006; Isaacowitz et al., 2006; Kim et al., 2008). The negative relation we find between age and past negative suggests that as people age they tend, on average, to look back on life with less regret. As Zimbardo and colleagues proposed, the present perspectives are the perspectives that mostly relate to affective states. Our findings of a negative relation between age and present hedonistic are consistent with what Carstensen and colleagues have found in their studies (Carstensen, 2006; Carstensen et al., 2011), that emotion regulation becomes a more important goal and one we are better equipped to pursue as we get older. According to our results, such regulation takes place through the present hedonistic view and not through the present fatalistic. Finally, regarding future time perspective, since what differs with age is the set of age-specific goals that people attend to, and not the future events, we did not find a relationship between age and future time perspective.

Future research could further explore the moderators that act as boundary conditions for our findings. For example, can key events in life shift time perspective? (Buss, 2009; Shipp and Cole, 2015) Does coping with stressful events affect time perspective differently at different ages or when the amount of life ahead is different? (Holman, 2015). Are there non-linear relations between age and time perspective in the late stages of life? Are there any different trajectories that mark changes in time perspective associated to the presence or absence of key events? (Palgi and Shmotkin, 2010). Future research may also explore other psychological mechanisms that may be consistent with our discussion of SST and SAVI models and complement our theorizing. Finally, assessing a “balanced” time perspective measure has been a central topic of interest and discussion, without absolute agreement on a unique way of measuring it (Stolarski et al., 2015b).

In conclusion, our results suggest that researchers should carefully consider the group of interest’s age in order to analyze age-related differences in the past negative and present hedonistic scores. Given that time perspective has a pervasive influence on human behavior, our results have broader implications to enhance the validity and reliability of psychological research.

## Author Contributions

DL-M and CT contributed to the design of the study, the theory development, data analysis and final write up. JU designed and conducted the literature search and participated in data analysis and final write up.

## Conflict of Interest Statement

The authors declare that the research was conducted in the absence of any commercial or financial relationships that could be construed as a potential conflict of interest.

## Footnotes

1 SST studies the perceived amount of time left in life. Since chronological age is negatively associated with the amount of time left in life, age-related patterns emerge. However, these patterns can be altered if individuals adopt a time perspective different from what is predicted by their place in the life cycle.

2 This type of attitude should not be confused with how much one enjoys life.

3 We conducted robustness checks using weighted linear regressions. The results were consistent with those from meta-regressions and ordinary regressions.
