# Supplementary material for: Time Perspective and Age: A Review of Age Associated Differences
Source: Front Psychol. 2017 Feb 17;8:101. doi: 10.3389/fpsyg.2017.00101 (PMC5313535; doi:10.3389/fpsyg.2017.00101)
Supplement: Supplementary file 1 [file Table_1.pdf]

Supplementary Table S1: Summary of studies included in the analyses

Paper	Context the ZTPI was conducted / Previous identify manipulations	# of studies in the paper	Country where the study was conducted	Descriptive characteristics of the population	N	Percentage of females	Age mean	Age S.D	ZTPI RESULTS									
									Mean PP*	Mean PN*	Mean PH*	Mean PF*	Mean F*	S.D. PP*	S.D PN*	S.D PH*	S.D PF*	S.D F*
(Jackson, Fritch, Nagasaka, & Pope, 2003)	Control setting / Procrastination scale	**1/2**	United States	Undergraduate students	147	0.71	21.55	5.76	3.61	3.05	3.40	2.40	3.37	0.61	0.72	0.48	0.63	0.51
		**2/2**			160	0.71	22.01	6.86	3.65	3.03	3.85	2.51	3.36	0.55	0.75	0.57	0.59	0.54
(Hamilton, Kives, Micevski, & Grace, 2003)	Control setting / none	**1/1**	Canada	Cardiac Rehabilitation	74	0.24	60.20	11.74	3.72	2.83	3.30	2.56	3.54	0.47	0.66	0.45	0.71	0.55
(Juan Francisco Díaz-Morales, 2006)	Control setting / none	**1/1**	Spain	General Population	756	0.62	40.10	N/A	3.29	2.64	3.05	2.75	3.56	0.30	0.30	0.30	0.30	0.30
(Henson, Carey, Carey, & Maisto, 2006)	Control setting / none	**1/1**	United States	Undergraduate and first year graduate students	1568	0.53	19.30	1.00	N/A	N/A	3.40	2.40	3.60	N/A	N/A	0.48	0.54	0.53
(Mello & Worrell, 2006)	Control setting / none	**1/1**	United States	Internship population	722	0.53	14.50	1.40	3.40	3.19	3.39	2.56	3.35	0.54	0.71	0.52	0.63	0.56
(Milfont & Gouveia, 2006)	Control setting / Values questionnaire	**1/1**	Brazil	Undergraduate	247	0.41	22.47	4.19	3.50	2.83	2.65	2.51	3.65	0.66	0.65	0.55	0.61	0.56
(Storm, 2006)	Control setting /Shapes which have an implication on mood	**1/1**	Australia	Students in general	200	0.62	22.00	7.00	N/A	N/A	3.60	2.68	3.33	N/A	N/A	0.52	0.58	0.56
(Ferrari & Díaz-Morales, 2007)	Snow ball approach in house / Procrastination scale	**1/1**	Spain	General Population	275	0.51	49.40	5.55	3.30	2.60	2.90	2.70	3.70	0.65	0.54	0.55	0.54	0.53
(Horstmanshof & Zimitat, 2007)	Control setting / none	**1/1**	Australia	Undergraduate and first year graduate students	347	0.65	22.00	N/A	3.53	3.27	3.43	2.58	3.40	0.55	0.63	0.42	0.55	0.49
(Livneh & Martz, 2007)	Control setting / none	**1/1**	United States	Diabetes patients with complications	106	0.66	48.90	14.40	3.59	3.07	3.34	2.62	3.47	0.75	0.73	0.61	0.67	0.52
(Sircova et al., 2007)	Control setting / none	**1/1**	Russia	General Population	1136	N/A	27.24	N/A	3.37	2.66	3.57	3.63	2.69	0.58	0.66	0.61	0.63	0.71
(Worrell & Mello, 2007)	Control setting / none	**1/1**	United States	Academically Talented Population	815	0.53	14.40	N/A	3.40	3.19	3.39	2.56	3.35	0.54	0.71	0.52	0.63	0.56
(J. F. Díaz-Morales, Ferrari, & Cohen, 2008)	Control setting / Procrastination scale	**1/1**	Spain	General Population	509	0.50	49.78	6.14	3.30	2.64	2.88	2.76	3.71	0.66	0.59	0.55	0.60	0.55
(Pluck et al., 2008)	Control setting / IQ test	**1/2**	United Kingdom	Homeless	50	0.16	33.40	7.55	3.05	3.73	3.59	3.21	3.02	0.65	0.60	0.48	0.66	0.49
		**2/2**		General Population	50	0.16	33.50	10.25	3.48	3.60	3.05	2.86	3.08	0.57	0.63	0.46	0.51	0.59
(U. do R. Leite & Luiz, 2008)	Control setting / none	**1/2**	Brazil	Students in general	1528	0.48	19.80	1.80	3.42	2.89	3.30	2.67	3.39	0.59	0.66	0.62	0.69	0.56
		**2/2**			1047	0.62	25.11	9.62	3.51	2.97	3.00	2.86	3.52	0.66	0.65	0.55	0.73	0.49
(Guthrie, Butler, & Ward, 2009)	At a hear salon / Health behaviors	**1/1**	United States	General Population	525	0.53	45.70	19.40	N/A	N/A	3.22	2.43	3.79	N/A	N/A	0.60	0.80	0.50
(Liniauskaite & Kairys, 2009)	Control setting / none	**1/4**	Lithuania	General Population	616	0.64	26.40	N/A	3.57	2.85	3.24	2.84	3.58	0.56	0.67	0.55	0.63	0.57
		**2/4**			625	0.41	35.28	N/A	3.57	2.85	3.24	2.84	3.58	0.56	0.67	0.55	0.63	0.57
		**3/4**			245	0.65	20.70	N/A	3.57	2.85	3.24	2.84	3.58	0.56	0.67	0.55	0.63	0.57
		**4/4**			40	0.90	19.80	N/A	3.57	2.85	3.24	2.84	3.58	0.56	0.67	0.55	0.63	0.57
(V. Ortuño & Gamboa,	Control setting / none	**1/1**	Portugal	Students in general	277	0.97	22.00	N/A	3.61	2.66	3.53	2.46	3.58	0.56	0.71	0.53	0.60	0.53

2009)																		
(Petkoska & Earl, 2009)	Online / none	**1/1**	Australia	Retirement population	366	0.54	54.00	3.50	3.65	2.64	3.22	2.50	3.55	0.54	0.73	0.49	0.65	0.46
(Boniwell, Osin, Alex Linley, & Ivanchenko, 2010)	Control setting / none	**1/2**	United Kingdom	Undergraduate	179	0.82	24.00	N/A	3.00	3.44	3.40	3.54	2.60	N/A	N/A	N/A	N/A	N/A
		**2/2**	Russia		289	0.54	22.00	N/A	2.51	3.38	3.58	3.67	2.49	N/A	N/A	N/A	N/A	N/A
(Daugherty & Brase, 2010)	Control setting /Money choice questionnaire	**1/1**	United States	Undergraduate and first year graduate students	467	0.63	18.99	1.54	N/A	N/A	3.58	2.54	3.59	N/A	N/A	0.48	0.57	0.50
(Luyckx, Lens, Smits, & Goossens, 2010)	Control setting / Identity style questionnaire	**1/2**	Belgium	First year graduate students	371	0.78	18.25	1.26	N/A	N/A	3.41	2.86	3.27	N/A	N/A	0.45	0.49	0.45
		**2/2**			371	0.78	18.25	1.26	N/A	N/A	3.36	2.78	3.36	N/A	N/A	0.44	0.49	0.46
(McElwee & Haugh, 2010)	Control setting / Clarity of future questionnaire	**1/1**	United States	Pre-undergraduate students	684	0.83	21.65	6.35	3.64	3.07	3.40	2.42	3.47	0.66	0.75	0.58	0.63	0.61
(Carelli, Wiberg, & Wiberg, 2011)	At home, sent questionnaire / none	**1/1**	Sweden	General Population	419	0.64	33.58	N/A	3.58	2.60	3.16	2.49	3.36	0.60	0.69	0.52	0.51	0.48
(Ely & Mercurio, 2011)	Control setting / none	**1/1**	United States	Undergraduate	230	0.51	19.10	1.60	3.80	3.00	N/A	N/A	3.40	0.60	0.70	N/A	N/A	0.60
(Protogerou & Turner-Cobb, 2011)	Control setting / none	**1/2**	United Kingdom	Students in general	93	0.71	20.00	3.03	N/A	N/A	3.52	2.84	3.36	N/A	N/A	0.60	0.55	0.42
		**2/2**	Greece		104	0.73	22.00	4.18	N/A	N/A	3.60	2.91	3.28	N/A	N/A	0.53	0.62	0.49
(Wittmann et al., 2011)	Control setting / Mindfulness questionnaire	**1/1**	United States	Undergraduate	27	0.44	21.10	2.20	N/A	N/A	3.65	2.33	3.43	N/A	N/A	0.43	0.57	0.48
(Zhang & Howell, 2011)	Control setting / Life satisfaction	**1/1**	United States	Undergraduate	754	0.70	25.02	9.16	3.50	3.06	3.36	2.51	3.49	0.59	0.71	0.53	0.63	0.51
(Anagnostopoulos & Griva, 2012)	Control setting / none	**1/2**	Greece	Students in general	337	0.67	20.80	N/A	3.39	2.61	3.50	2.26	3.50	0.61	0.69	0.59	0.57	0.64
		**2/2**			120	0.59	28.82	N/A	3.39	2.61	3.50	2.26	3.50	0.61	0.69	0.59	0.57	0.64
(Baumann & Odum, 2012)	Control setting / none	**1/1**	United States	Undergraduate and first year graduate students	143	0.59	19.88	2.40	3.74	2.94	3.40	2.23	3.47	0.53	0.67	0.48	0.52	0.48
(Cretu, 2012)	Control setting / none	**1/1**	Romania	Romanian ethnics	1260	0.63	30.66	N/A	4.49	3.32	4.69	3.39	5.55	1.63	1.83	1.58	1.38	1.35
(Desmyter & De Raedt, 2012)	At home / none	**1/1**	Belgium	General Population	149	0.58	75.50	7.00	3.48	2.89	3.06	3.11	3.37	0.44	0.69	0.52	0.54	0.42
(Gruber, Cunningham, Kirkland, & Hay, 2012)	Online / none	**1/2**	United States	Undergraduate	509	0.59	19.35	4.32	3.53	2.95	3.29	2.43	3.46	0.61	0.66	0.61	0.62	0.59
		**2/2**			509	0.59	19.35	4.32	3.10	3.28	3.50	2.68	3.24	0.58	0.71	0.66	0.57	0.60
(Ishino & Shimizu, 2012)	Control setting / Personal important and dream questionnaire	**1/1**	Japan	Undergraduate	309	0.41	19.71	0.81	3.46	3.62	3.61	3.09	2.62	0.74	0.86	0.58	0.65	0.67
(Merson, Guillon, Arvers, Underner, & Perriot, 2012)	Control setting / Social deprivation scale	**1/2**	France	Smoking cessation people	192	0.39	45.60	N/A	2.80	3.00	2.70	2.80	3.10	N/A	N/A	N/A	N/A	N/A
		**2/2**			192	0.46	47.00	N/A	3.10	2.40	3.10	2.20	3.50	N/A	N/A	N/A	N/A	N/A
(McElheran, 2012)	Online / none	**1/1**	Canada	General population	288	0.79	37.02	11.83	3.76	2.74	3.34	2.24	3.61	0.68	0.74	0.50	0.58	0.50
(Bolotova & Hachaturova, 2013)	Control setting / none	**1/1**	Russia	General Population	295	0.53	32.00	N/A	3.74	2.47	3.42	2.51	3.71	0.59	0.74	0.54	0.65	0.55
(Chittaro & Vianello, 2013)	Online / none	**1/1**	Online population	Facebook users	149	0.47	32.40	11.80	3.38	3.22	3.46	2.69	3.30	0.57	0.69	0.47	0.56	0.45

(Cinan & Doğan, 2013)	Control setting / none	**1/1**	Turkey	Undergraduate	110	0.76	20.11	3.80	3.74	2.98	3.64	2.57	3.68	0.65	0.78	0.59	0.62	0.58
(Daniel, Stanton, & Epstein, 2013)	Online / none	**1/2**	United States	Specialize population	48	1.00	23.59	3.50	3.69	3.10	3.36	2.63	3.70	0.45	0.76	0.42	0.59	0.43
		**2/2**			48	1.00	26.22	5.86	3.80	3.15	3.33	2.64	3.62	0.64	0.59	0.52	0.57	0.47
(Griva, Anagnostopoulos, & Potamianos, 2013)	Breast cancer doctor's office / none	**1/1**	Greece	Woman in breast cancer sessions	194	1.00	51.88	11.00	3.45	2.53	2.86	2.50	3.42	N/A	N/A	N/A	N/A	N/A
(King & Gaerlan, 2013)	Control setting / none	**1/1**	Philippines	First year graduate students	385	0.45	17.66	0.97	3.67	3.50	3.72	2.92	3.41	0.41	0.57	0.40	0.56	0.42
(Nordhall & Agerström, 2013)	Control setting / Moral values questionnaire	**1/2**	Sweden	General Population	132	0.59	25.57	8.00	N/A	N/A	3.40	2.60	3.29	N/A	N/A	0.48	0.54	0.52
		**2/2**			119	0.78	26.20	9.00	N/A	N/A	3.40	2.60	3.29	N/A	N/A	0.48	0.54	0.52
(V. E. C. Ortuño, Paixão, & Nunes Janeiro, 2013)	Control setting / none	**1/2**	Portugal	Graduate students	277	0.63	22.06	5.40	3.65	2.61	3.57	2.41	3.69	0.52	0.61	0.44	0.53	0.50
		**2/2**			264	0.92	19.44	2.85	3.65	2.61	3.57	2.41	3.69	0.52	0.61	0.44	0.53	0.50
(Stolarski, Ledzińska, & Matthews, 2013)	Control setting / Morning - Evening questionnaire	**1/2**	Poland	Students in general	309	0.70	22.50	3.50	3.60	2.81	3.41	2.40	3.59	0.60	0.72	0.55	0.60	0.57
	Control setting / Carrier decision making difficulties	**2/2**	United States	Carrier counseling	195	0.60	39.85	11.64	3.40	3.10	3.10	2.40	3.70	0.62	0.72	0.51	0.53	0.51
(Cheong, Tucker, Simpson, & Chandler, 2014)	Control setting / Substance use	**1/1**	United States	African American youth living in disadvantaged urban areas	344	0.68	18.86	5.00	3.69	3.10	3.60	2.69	3.64	0.58	0.76	0.51	0.69	0.51
(Incollingo Belsky, Epel, & Tomiyama, 2014)	Control setting / Food diaries for 4 weeks	**1/1**	United States	People in a weight lose treatment	65	0.22	54.95	14.36	3.89	2.67	3.27	2.53	3.62	0.48	0.48	0.55	0.68	0.45
(McKay, Worrell, Temple, Perry, & Cole, 2014)	Control setting / none	**1/3**	United Kingdom	Pre-undergraduate students	2171	0.50	13.50	2.00	3.83	2.97	3.67	2.64	3.36	0.78	1.06	0.74	0.74	0.87
		**2/3**	United States	Academically talented students	2171	0.53	14.50	3.00	3.49	2.86	3.60	2.40	3.48	0.76	1.07	0.71	0.76	0.80
		**3/3**	Australia	General Population	2171	0.68	43.50	10.00	3.81	2.93	3.38	2.45	3.60	0.63	1.00	0.65	0.68	0.07
(Oyanadel & Buela-Casal, 2014)	Work place / none	**1/1**	Chile	People receiving treatment for different health conditions	167	0.70	42.38	12.25	3.26	3.66	3.31	2.36	0.86	1.04	0.99	0.90	0.91	0.86
(Oyanadel, 2014)	Control setting / none	**1/1**	Chile	General population	604	0.59	29.90	12.50	3.69	2.85	3.39	2.80	3.54	N/A	N/A	N/A	N/A	N/A
(Leite, 2014)	Control setting / none	**1/1**	Brazil	General Population	1965	0.60	24.93	14.40	3.54	2.98	3.45	2.79	3.48	N/A	N/A	N/A	N/A	N/A

Table legend:

*

PP	Past Positive
PN	Past Negative
PH	Present Hedonistic
PF	Present Fatalistic
F	Future
S.D.	Standard Deviation
