# Supplementary figures and images for: Time Perspective and Age: A Review of Age Associated Differences
Source: Front Psychol. 2017 Feb 17;8:101. doi: 10.3389/fpsyg.2017.00101 (PMC5313535; doi:10.3389/fpsyg.2017.00101)

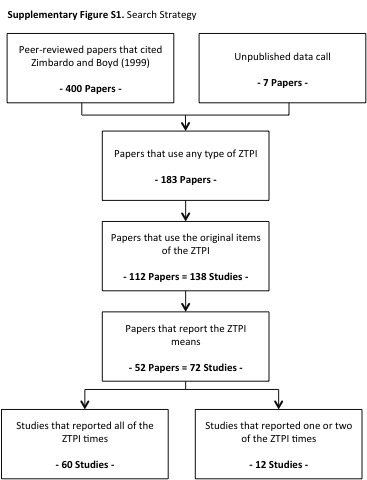

Supplement: Supplementary file 2 [file Image_1.jpg]

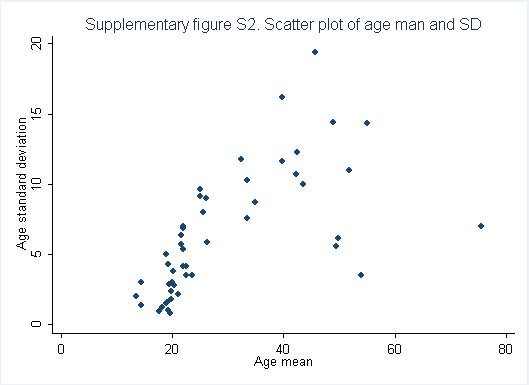

Supplement: Supplementary file 3 [file Image_2.tif]

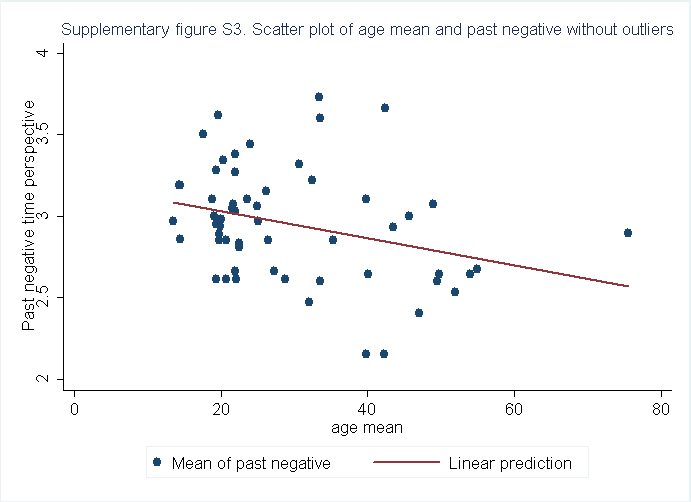

Supplement: Supplementary file 4 [file Image_3.tif]

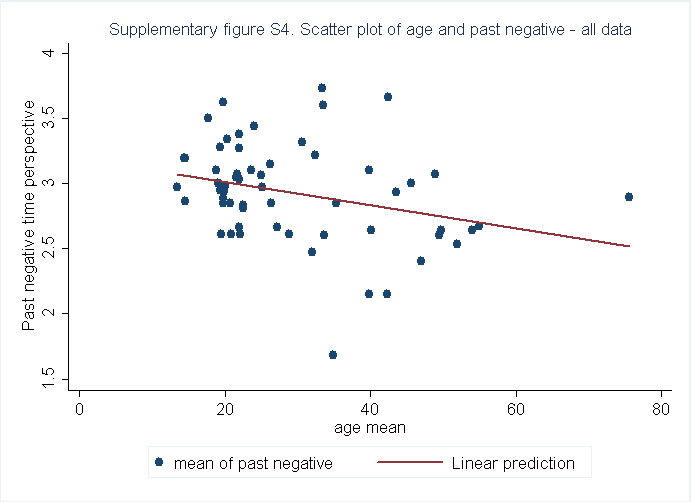

Supplement: Supplementary file 5 [file Image_4.tif]

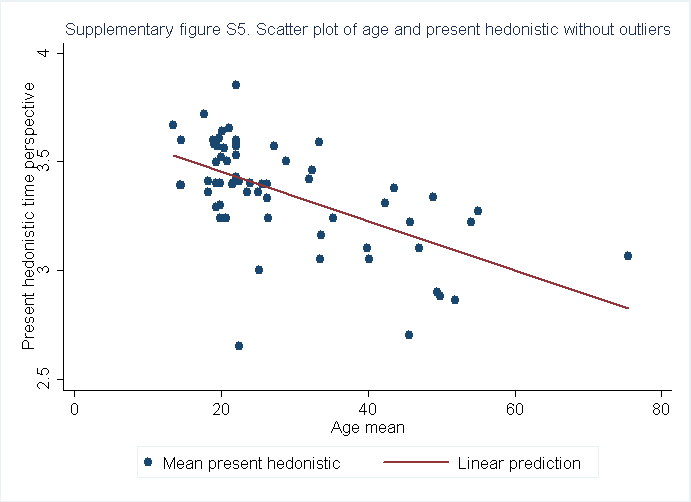

Supplement: Supplementary file 6 [file Image_5.tif]

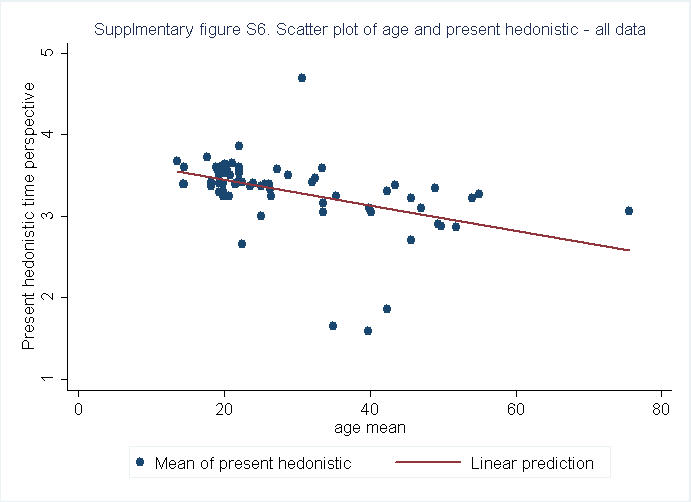

Supplement: Supplementary file 7 [file Image_6.tif]
